# Cost-effectiveness of pembrolizumab for advanced non-small cell lung cancer patients with varying comorbidity burden

**DOI:** 10.1371/journal.pone.0228288

**Published:** 2020-01-29

**Authors:** Steven D. Criss, Lauren Palazzo, Tina R. Watson, Adelle M. Paquette, Keith Sigel, Juan Wisnivesky, Chung Yin Kong

**Affiliations:** 1 Institute for Technology Assessment, Massachusetts General Hospital, Boston, MA, United States of America; 2 Division of General Internal Medicine, Icahn School of Medicine at Mount Sinai, New York, NY, United States of America; 3 Division of Pulmonary and Critical Care Medicine, Icahn School of Medicine at Mount Sinai, New York, NY, United States of America; 4 Harvard Medical School, Boston, MA, United States of America; Universidade do Algarve Departamento de Ciencias Biomedicas e Medicina, PORTUGAL

## Abstract

**Objectives:**

While previous cost-effectiveness studies on pembrolizumab in stage IV non-small cell lung cancer (NSCLC) have found these regimens to be cost-effective, their reliance on randomized controlled trial (RCT) data with strict inclusion criteria limits generalizability to patients with comorbidities. We estimated the cost-effectiveness of first-line pembrolizumab for patients with various comorbidities.

**Materials and methods:**

In our base case analysis, we studied pembrolizumab plus chemotherapy (pembrolizumab combination therapy) versus chemotherapy alone. In a secondary analysis, we considered only patients with PD-L1 expression of at least 50% (PD-L1-high) and evaluated pembrolizumab monotherapy, pembrolizumab combination therapy, and chemotherapy alone. Microsimulation models were developed for the base case and the PD-L1-high analyses. To estimate outcomes of patients with differing comorbidities, we combined survival data from patients with few or no comorbidities from the RCTs with estimates from the general population obtained from the Surveillance, Epidemiology, and End Results (SEER)-Medicare database. Comorbidity burden level was divided into three groups based on the Charlson score (equal to 0, 1, or 2+); patients with various other specific comorbidities were also analyzed. Incremental cost-effectiveness ratios (ICER) were compared to a willingness-to-pay (WTP) threshold of $100,000/quality-adjusted life-year (QALY).

**Results:**

In the Charlson 0, Charlson 1, and Charlson 2+ patient populations, estimated ICERs for pembrolizumab combination therapy in the base case model were $173,919/QALY, $175,165/QALY, and $181,777/QALY, respectively, compared to chemotherapy.

In the PD-L1-high model, the Charlson 0, Charlson 1, and Charlson 2+ patients had ICERs of $147,406/QALY, $149,026/QALY, and $154,521/QALY with pembrolizumab combination therapy versus chemotherapy. Pembrolizumab monotherapy was weakly dominated for each comorbidity group in the PD-L1-high model.

**Conclusion:**

For patients with stage IV NSCLC and varying comorbidity burden, first-line treatment with pembrolizumab does not represent a cost-effective strategy compared to chemotherapy. Resources should be focused on collecting immunotherapy survival data for more representative NSCLC patient populations.

## Introduction

Recent advancements in the treatment of non-small cell lung cancer (NSCLC) have aimed to extend survival for patients with the poorest prognoses—those with advanced metastatic disease. With an estimated 154,000 deaths in the U.S. due to lung cancer in 2018 and a five-year survival rate of 5% for those with distant metastases[1], the pursuit of improved late-stage survival is an essential one. Over the last several years, randomized controlled trials (RCT) for immune checkpoint inhibitors in the first-line treatment of stage IV NSCLC have demonstrated significant clinical efficacy [2–7], leading to swift, widespread adoption of their use. However, concern has arisen regarding the high prices of these therapies, as nearly 40% of approximately 200,000 NSCLC patients were expected to be diagnosed with stage IV disease in 2018 [8, 9].

Of the immunotherapy treatments currently approved by the U.S. Food and Drug Administration for first-line use, pembrolizumab in combination with chemotherapy for both nonsquamous and squamous patients, as well as pembrolizumab monotherapy for patients with high levels of programmed death ligand-1 (PD-L1) expression have been studied using cost-effectiveness analyses [10–13]. Based on survival data from their respective RCTs, these treatment regimens have been estimated to be cost-effective compared to standard-of-care chemotherapy [10–13]. Of note, however, is that the sole reliance on RCT data for these evaluations has excluded populations, such as persons with major comorbid illnesses, that typically fall outside of the strict inclusion criteria of RCTs. An analysis of the SWOG historical database, a national clinical trials consortium sponsored by the National Cancer Institute, found that approximately 60% of exclusion criteria for cancer treatment clinical trials were found to be related to comorbidity or performance status [14]. Moreover, the presence of one or more comorbidities has been associated with decreased discussions about enrolling in, offers to enroll in, and participation in a clinical trial for cancer treatment [15].

The bias toward enrolling healthier patients in RCTs could have implications for the generalizability of cost-effectiveness analyses to the actual population of lung cancer patients in the U.S. As low as 0.8% of lung cancer patients enroll in clinical trials and real-world data indicate that survival may be shorter than reported in clinical trials [16, 17], calling into question how representative RCTs could be of the broader lung cancer patient population, of whom more than half have major comorbidities [18–20]. In this study, we estimated the cost-effectiveness of first-line pembrolizumab for patients with varying levels and types of comorbidities using a novel modeling paradigm that combines clinical trial and real-world patient data.

## Materials and methods

### Simulation models

We developed microsimulation models to estimate the cost-effectiveness of pembrolizumab treatment regimens for stage IV NSCLC patients with varying burden and types of comorbidity from the healthcare sector perspective. In our base case analysis, we evaluated pembrolizumab plus chemotherapy (pembrolizumab combination therapy) versus chemotherapy alone. In a secondary analysis, we considered a patient population with PD-L1 expression of at least 50% (PD-L1-high) and evaluated pembrolizumab monotherapy, pembrolizumab combination therapy, and chemotherapy alone as competing choices. To ensure stable simulation outcomes, we simulated one million patients as they transitioned from first-line treatment to second-line treatment, and ultimately to death. Patients were followed on a monthly basis through each of these health states, using a 3% annual discount rate for survival and cost projections [21]. We calculated incremental cost-effectiveness ratios (ICERs) for each strategy and compared them to a willingness-to-pay (WTP) threshold of $100,000/quality-adjusted life-year (QALY) [22].

**Fig 1:**
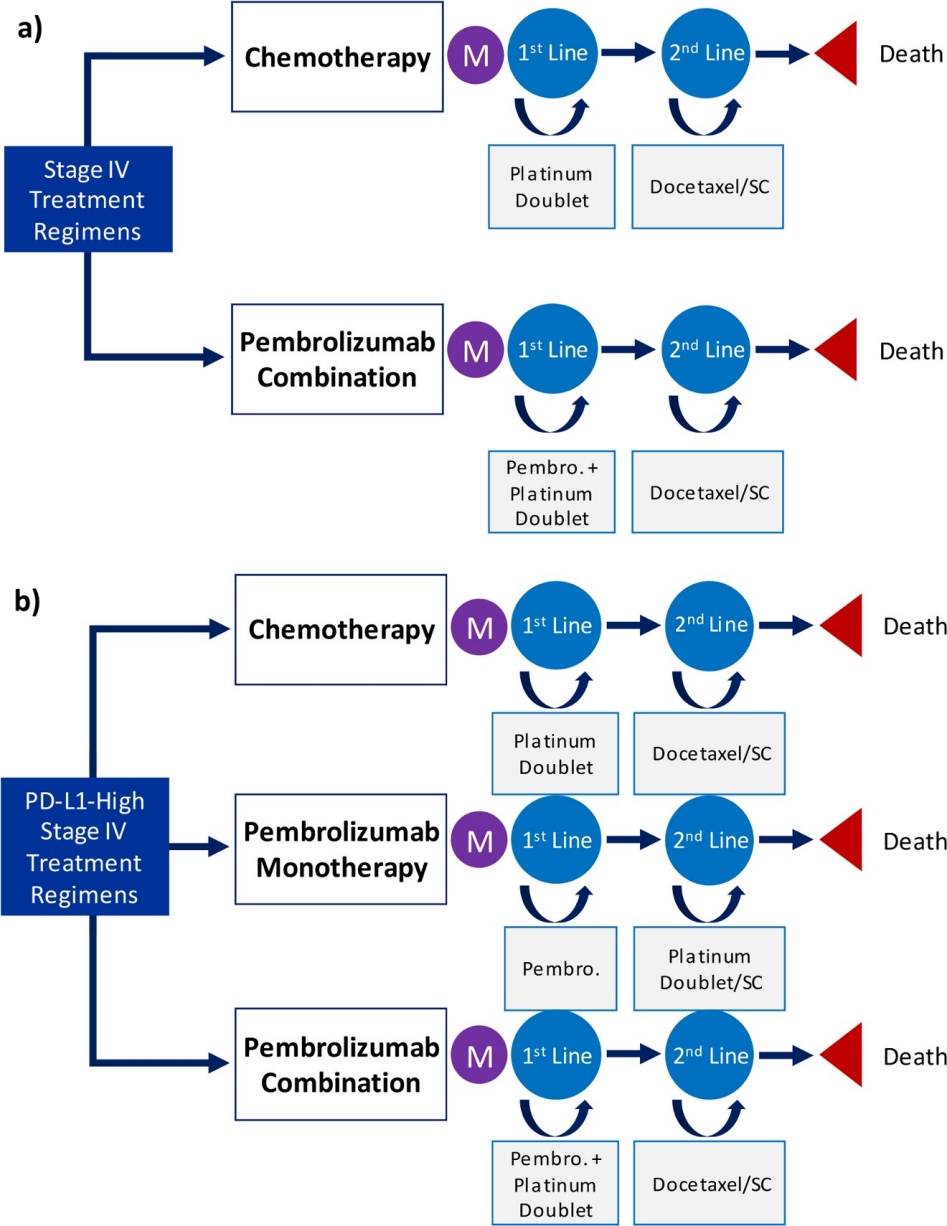
Summary schematic of model structures. a)Pembrolizumab combination therapy model b)Pembrolizumab for PD-L1-high patients model M, Markov node; 1^st^ Line, first-line treatment; 2^nd^ Line, second-line treatment; Pembro., pembrolizumab; SC, supportive care.

General structures for the models from each analysis are summarized in Fig 1. In both treatment models, the first-line chemotherapy arm did not differentiate between cisplatin-based and carboplatin-based treatments, as these drugs are commonly used in similar capacities in clinical practice because of their similar survival outcomes and comparably low cost [23–25]. In addition to platinum-based chemotherapies, nonsquamous patients were given pemetrexed and squamous patients were given paclitaxel (40% received nanoparticle albumin-bound (nab)–paclitaxel) until progression. After progression to second-line treatment in the chemotherapy arm, patients were treated with docetaxel until further progression, at which point supportive care was assumed to be the only treatment provided.

The pembrolizumab treatment arms followed the administration specifications of their respective RCTs [2–5]. In the pembrolizumab combination therapy arms, first-line treatment included pembrolizumab given concurrently with platinum-based chemotherapy plus pemetrexed or paclitaxel (40% received nab–paclitaxel), depending on histology. In second-line treatment, patients were treated with docetaxel and then supportive care only. In the pembrolizumab monotherapy arm, first-line treatment consisted of pembrolizumab alone. In second-line treatment, patients were treated with platinum-based chemotherapy plus pemetrexed or paclitaxel (40% received nab–paclitaxel), then with supportive care only. Additional information on dosing and therapy infusion intervals can be found in Table A S1 File.

### Data source and study population

To estimate the outcomes of patients with differing comorbidity burden, we combined the survival results of patients with few or no comorbidities enrolled in RCTs with general population data from the Surveillance, Epidemiology, and End Results (SEER)-Medicare database using a competing risk survival analysis method (described in “2.3 Modeling Survival” section) [26]. From the SEER-Medicare data, we selected patients age 65 or older diagnosed between 1991 and 2011 with stage IV NSCLC as their only cancer. Histology was identified with International Classification of Diseases for Oncology (ICD-O-3) codes and histological categories were determined according to the SEER Cancer Statistics Review [27]. We further selected patients who received first-line carboplatin-based or cisplatin-based chemotherapy (comparable to the chemotherapy regimens given in both RCTs) [2–4, 28]. Instances of receiving carboplatin-based or cisplatin-based chemotherapy were identified in Medicare claims data using relevant Healthcare Common Procedure Coding System codes. The first date corresponding to a chemotherapy claim was defined as the beginning of first-line treatment and the last date corresponding to such a claim plus the typical three-week dosing cycle was defined as the end of first-line treatment. Our study cohort was comprised of 16,492 lung cancer patients (of whom 24.5% had squamous histology) receiving these treatments. Summary statistics for the SEER-Medicare data are provided in Table B S1 File.

The next section outlines our method of modeling the survivals of patients with differing levels of comorbidity, measured by the Charlson score, as well as with specific conditions. Charlson score was calculated based on comorbidities present in the year before cancer diagnosis, using the Comorbidity SAS Macro provided by National Cancer Institute. Comorbidity burden level was divided into three groups—Charlson score equal to 0 (Charlson 0), Charlson score equal to 1 (Charlson 1), and Charlson score equal to 2+ (Charlson 2+). Additionally, we evaluated specific comorbidities that are relatively prevalent in lung cancer patients, including peripheral vascular disease, diabetes, chronic obstructive pulmonary disease, cerebrovascular disease, and congestive heart failure.

### Modeling survival

Our analysis of the SEER-Medicare database was used to procure time on treatment (ToT) data and survival estimates for patients who received first-line carboplatin-based or cisplatin-based standard-of-care chemotherapy [28]. The median ToT was used to estimate the rate of progression from first-line treatment to second-line treatment. Survival estimates were derived using a competing risks analysis framework [26]. Cumulative incidence curves stratified by histology and comorbidity level or type were generated for lung cancer-specific versus other-cause mortality. Cause-specific survival curves were then derived from the incidence functions and used to estimate monthly death rates. Lung cancer-specific death rates were based on monthly rate estimates from the survival curves for the first three years, with rates derived from exponential functions fitted to the survival curves thereafter. Other-cause death rates were also modeled using exponential functions. The monthly rates for lung cancer-specific death and other-cause death were then summed to produce the overall survival (OS) rate for chemotherapy patients.

In order to estimate the lung cancer-specific death rate for patients treated with the pembrolizumab regimens, we multiplied the hazard ratios for death (pembrolizumab treatment versus chemotherapy treatment) from the respective pembrolizumab RCTs to the lung cancer-specific death rates determined for chemotherapy patients from SEER-Medicare data [2–4, 28]. The resulting lung cancer-specific death rates represent the estimated death rates of patients with comorbidities had they been treated with the pembrolizumab regimens. This adjusted rate was then added to the other-cause death rate to derive OS rates for pembrolizumab-treated patients. The OS rates were then used to evaluate the effectiveness of pembrolizumab combination therapy and pembrolizumab monotherapy versus chemotherapy in patients with differing comorbidity burden.

### Health state utilities

Quality of life utilities used in calculating QALYs were sourced from the literature [29, 30]. QALYs were estimated by adjusting life-years by utilities for age and phase of cancer progression. We assumed a 20% improvement in quality of life utility for those patients on pembrolizumab monotherapy, given its superior toxicity profile compared to chemotherapy. Additional information on health state utilities is provide in Table C S1 File.

### Costs

Direct medical expenses related to cancer treatment in our analysis were taken from relevant U.S. sources and included drug acquisition, therapy administration, treatment of major adverse events, follow-up scans, immunohistochemical testing, monthly supportive care, and death-related costs [31–34]. Unit drug costs are based on the Centers for Medicare & Medicaid Services (CMS) April 2019 Average Sales Price Drug Pricing Files (version updated March 22, 2019) [31]. For dosing considerations, body surface area was assumed to be 1.79 square meters, based on an analysis of an internal database containing over 3,500 lung cancer patients treated at Partners Healthcare hospitals. We considered treatment costs for adverse events that were rated at least grade 3 or higher [2, 4, 5]. Treatment costs for these adverse events were based on estimates by the Agency for Healthcare Research and Quality’s Healthcare Cost and Utilization Project using relevant International Classification of Diseases (ICD-9) codes [33]. Cost parameters used in the model are detailed in Table C S1 File. Costs were adjusted to 2019 U.S. dollars using the Personal Healthcare Price Index published by the CMS [35, 36].

### Sensitivity analysis

We performed one-way deterministic sensitivity analyses on model inputs to determine how uncertainty may affect the results. For each treatment model, 95% confidence intervals or plausible ranges were used to test the models at the upper and lower limits of each input parameter (Table 1). Deterministic sensitivity analyses were performed in the Charlson 0 patient population as a reference case and the results for variables with the 10 largest magnitudes of effect are shown.

**Table 1:** Deterministic sensitivity analysis variable ranges.

Variables	Estimate	Lower	Upper
**Utilities**			
Stage IV NSCLC (with chemotherapy) ^a^	0.76	0.57	0.95
Stage IV NSCLC (without chemotherapy) ^a^ ^b^	0.91	0.68	1.00
Age 69 years & younger (Avg. Male & Female) ^a^ ^b^	0.83	0.62	1.00
Age 70–79 years (Avg. Male & Female) ^a^ ^b^	0.80	0.60	1.00
Age 80 years & older (Avg. Male & Female) ^a^	0.75	0.56	0.94
**Costs**			
Pembrolizumab Price/mg ^a^	$49.20	$36.90	$61.50
Pemetrexed Price/mg ^a^	$6.83	$5.12	$8.54
Carboplatin Price/mg ^a^	$0.06	$0.05	$0.08
Paclitaxel Price/mg ^a^	$0.15	$0.11	$0.19
Nab-Paclitaxel (protein bound) Price/mg ^a^	$11.85	$8.89	$14.81
Docetaxel Price/mg ^a^	$1.16	$0.87	$1.45
Best Supportive Care Cost (regression estimate for 70 year old) ^a^	$637	$478	$796
Death Cost (regression estimate for 70 year old) ^a^	$9,433	$7,075	$11,791
**Survival**			
First-line chemotherapy ToT (nonsquamous, months) ^a^	2.76	2.07	3.45
First-line chemotherapy ToT (squamous, months) ^a^	2.76	2.07	3.45
First-line pembrolizumab combination ToT (nonsquamous, months) ^a^	7.40	5.55	9.25
First-line pembrolizumab combination ToT (squamous, months) ^a^	6.30	4.73	7.88
First-line pembrolizumab monotherapy ToT (months) ^a^	7.00	5.25	8.75
HR for death pembrolizumab combination (nonsquamous)	0.49	0.38	0.64
HR for death pembrolizumab combination (nonsquamous, PD-L1≥50%)	0.42	0.26	0.68
HR for death pembrolizumab combination (squamous)	0.64	0.49	0.85
HR for death pembrolizumab combination (squamous, PD-L1≥50%)	0.64	0.37	1.10
HR for death pembrolizumab monotherapy (PD-L1≥50%)	0.63	0.47	0.86
**Other**			
Body Surface Area (meters^2^)	1.79	1.78	1.80

^a^ range indicates 25% change

^b^ Maximum utility of 1.00

NSCLC, non-small cell lung cancer; ToT, time on treatment; LC, lung cancer; prog., progression; HR, hazard ratio

## Results

### Pembrolizumab combination therapy versus chemotherapy alone

In Charlson 0 patients, treating with chemotherapy alone in the first line resulted in a mean cost per patient of $53,805 and mean quality-adjusted survival of 0.67 QALYs. The pembrolizumab combination therapy regimen produced a mean cost per patient of $159,026 and mean quality-adjusted survival of 1.27 QALYs, for an ICER of $173,919/QALY compared to the chemotherapy regimen. In Charlson 1 patients, treating patients with chemotherapy alone resulted in a mean cost per patient of $52,905 and mean quality-adjusted survival of 0.66 QALYs. Treating with pembrolizumab combination therapy resulted in a mean cost per patient of $156,544 and mean quality-adjusted survival of 1.26 QALYs, providing an ICER of $175,165/QALY compared to chemotherapy alone. In Charlson 2+ patients, the chemotherapy regimen produced a mean cost per patient of $50,818 and mean quality-adjusted survival of 0.64 QALYs. The pembrolizumab combination therapy regimen resulted in a mean cost per patient of $153,825 and mean quality-adjusted survival of 1.20, for an ICER of $181,777/QALY.

Table 2 provides additional information for these results, as well as summary results for the analysis of these treatment strategies in patients with particular comorbidities, including peripheral vascular disease, diabetes, chronic obstructive pulmonary disease, cerebrovascular disease, and congestive heart failure. The lowest ICER comparing pembrolizumab combination therapy to chemotherapy alone occurred in the patient population with peripheral vascular disease ($174,775/QALY), while the highest ICER occurred in the patient population with congestive heart failure ($185,651/QALY).

**Table 2:** Summary results for patients in the pembrolizumab combination therapy model.

**Charlson 0**					
Strategy	Cost	QALY	Inc. Cost	Inc. QALY	ICER
Chemotherapy	$53,805	0.67	-	-	-
Pembro. Comb.	$159,026	1.27	$105,221	0.61	$173,919
**Charlson 1**					
Strategy	Cost	QALY	Inc. Cost	Inc. QALY	ICER
Chemotherapy	$52,905	0.66	-	-	-
Pembro. Comb.	$156,544	1.26	$103,639	0.59	$175,165
**Charlson 2+**					
Strategy	Cost	QALY	Inc. Cost	Inc. QALY	ICER
Chemotherapy	$50,818	0.64	-	-	-
Pembro. Comb.	$153,825	1.20	$103,007	0.57	$181,777
**Peripheral Vascular Disease**					
Strategy	Cost	QALY	Inc. Cost	Inc. QALY	ICER
Chemotherapy	$52,210	0.67	-	-	-
Pembro. Comb.	$155,764	1.26	$103,554	0.59	$174,775
**Diabetes**					
Strategy	Cost	QALY	Inc. Cost	Inc. QALY	ICER
Chemotherapy	$52,274	0.64	-	-	-
Pembro. Comb.	$155,252	1.22	$102,978	0.58	$177,803
**Chronic Obstructive Pulmonary Disease**					
Strategy	Cost	QALY	Inc. Cost	Inc. QALY	ICER
Chemotherapy	$52,498	0.66	-	-	-
Pembro. Comb.	$155,272	1.23	$102,774	0.58	$178,737
**Cerebrovascular Disease**					
Strategy	Cost	QALY	Inc. Cost	Inc. QALY	ICER
Chemotherapy	$50,308	0.62	-	-	-
Pembro. Comb.	$152,966	1.18	$102,658	0.56	$183,046
**Congestive Heart Failure**					
Strategy	Cost	QALY	Inc. Cost	Inc. QALY	ICER
Chemotherapy	$48,962	0.61	-	-	-
Pembro. Comb.	$150,760	1.16	$101,799	0.55	$185,651

QALY, quality-adjusted life-year; Inc., incremental; ICER, incremental cost-effectiveness ratio

Pembro. Comb., pembrolizumab combination therapy

### Pembrolizumab treatments versus chemotherapy alone for PD-L1-high patients

In the PD-L1-high model, pembrolizumab combination therapy weakly dominated pembrolizumab monotherapy in each scenario compared to chemotherapy alone, regardless of comorbidity level or type. Results for all strategies by comorbidity level and type are summarized in Table 3.

**Table 3:** Summary results for patients in the PD-L1-high model.

**Charlson 0**					
Strategy	Cost	QALY	Inc. Cost	Inc. QALY	ICER
Chemotherapy	$53,870	0.67	-	-	-
Pembro. Mono.	$138,123	1.14	Weakly Dominated		
Pembro. Comb.	$164,547	1.42	$110,677	0.75	$147,406
**Charlson 1**					
Strategy	Cost	QALY	Inc. Cost	Inc. QALY	ICER
Chemotherapy	$52,966	0.66	-	-	-
Pembro. Mono.	$134,243	1.12	Weakly Dominated		
Pembro. Comb.	$161,879	1.40	$108,913	0.73	$149,026
**Charlson 2+**					
Strategy	Cost	QALY	Inc. Cost	Inc. QALY	ICER
Chemotherapy	$50,997	0.64	-	-	-
Pembro. Mono.	$131,700	1.07	Weakly Dominated		
Pembro. Comb.	$159,677	1.34	$108,680	0.70	$154,521
**Peripheral Vascular Disease**					
Strategy	Cost	QALY	Inc. Cost	Inc. QALY	ICER
Chemotherapy	$52,308	0.67	-	-	-
Pembro. Mono.	$133,918	1.12	Weakly Dominated		
Pembro. Comb.	$161,530	1.40	$109,223	0.73	$149,450
**Diabetes**					
Strategy	Cost	QALY	Inc. Cost	Inc. QALY	ICER
Chemotherapy	$52,371	0.64	-	-	-
Pembro. Mono.	$133,268	1.09	Weakly Dominated		
Pembro. Comb.	$161,209	1.36	$108,837	0.72	$151,513
**Chronic Obstructive Pulmonary Disease**					
Strategy	Cost	QALY	Inc. Cost	Inc. QALY	ICER
Chemotherapy	$52,622	0.66	-	-	-
Pembro. Mono.	$132,652	1.10	Weakly Dominated		
Pembro. Comb.	$160,698	1.37	$108,075	0.71	$152,040
**Cerebrovascular Disease**					
Strategy	Cost	QALY	Inc. Cost	Inc. QALY	ICER
Chemotherapy	$50,415	0.62	-	-	-
Pembro. Mono.	$130,690	1.06	Weakly Dominated		
Pembro. Comb.	$158,308	1.31	$107,894	0.69	$155,991
**Congestive Heart Failure**					
Strategy	Cost	QALY	Inc. Cost	Inc. QALY	ICER
Chemotherapy	$49,079	0.61	-	-	-
Pembro. Mono.	$127,567	1.03	Weakly Dominated		
Pembro. Comb.	$156,476	1.28	$107,396	0.67	$159,697

QALY, quality-adjusted life-year; Inc., incremental; ICER, incremental cost-effectiveness ratio

Pembro. Mono., pembrolizumab monotherapy

In Charlson 0 patients, providing chemotherapy alone in the first line resulted in a mean cost per patient of $53,870 and mean quality-adjusted survival of 0.67 QALYs. The pembrolizumab combination therapy regimen resulted in a mean cost per patient of $164,547, with mean quality-adjusted survival of 1.42 QALYs, giving an ICER of $147,406/QALY compared to chemotherapy alone. In Charlson 1 patients, treatment with chemotherapy resulted in a mean cost per patient of $52,966 and mean quality-adjusted survival of 0.66 QALYs. Pembrolizumab combination therapy resulted in a mean cost per patient of $161,879 and mean quality-adjusted survival of 1.40 QALYs, for an ICER of $149,026/QALY compared to chemotherapy alone. Finally, in the Charlson 2+ population, treating with chemotherapy resulted in a mean cost per patient of $50,997 and mean quality-adjusted survival of 0.64 QALYs. Treating with pembrolizumab combination therapy resulted in a mean cost per patient of $159,677 and mean quality-adjusted survival of 1.34 QALYs, producing an ICER of $154,521/QALY compared to chemotherapy.

The ICER comparing pembrolizumab combination therapy to chemotherapy alone was lowest in the patient population with peripheral vascular disease ($149,450/QALY) and highest in the patient population with congestive heart failure ($159,697/QALY), showing a relatively narrow range for ICERs by comorbidity.

### Sensitivity analysis

**Fig 2:**
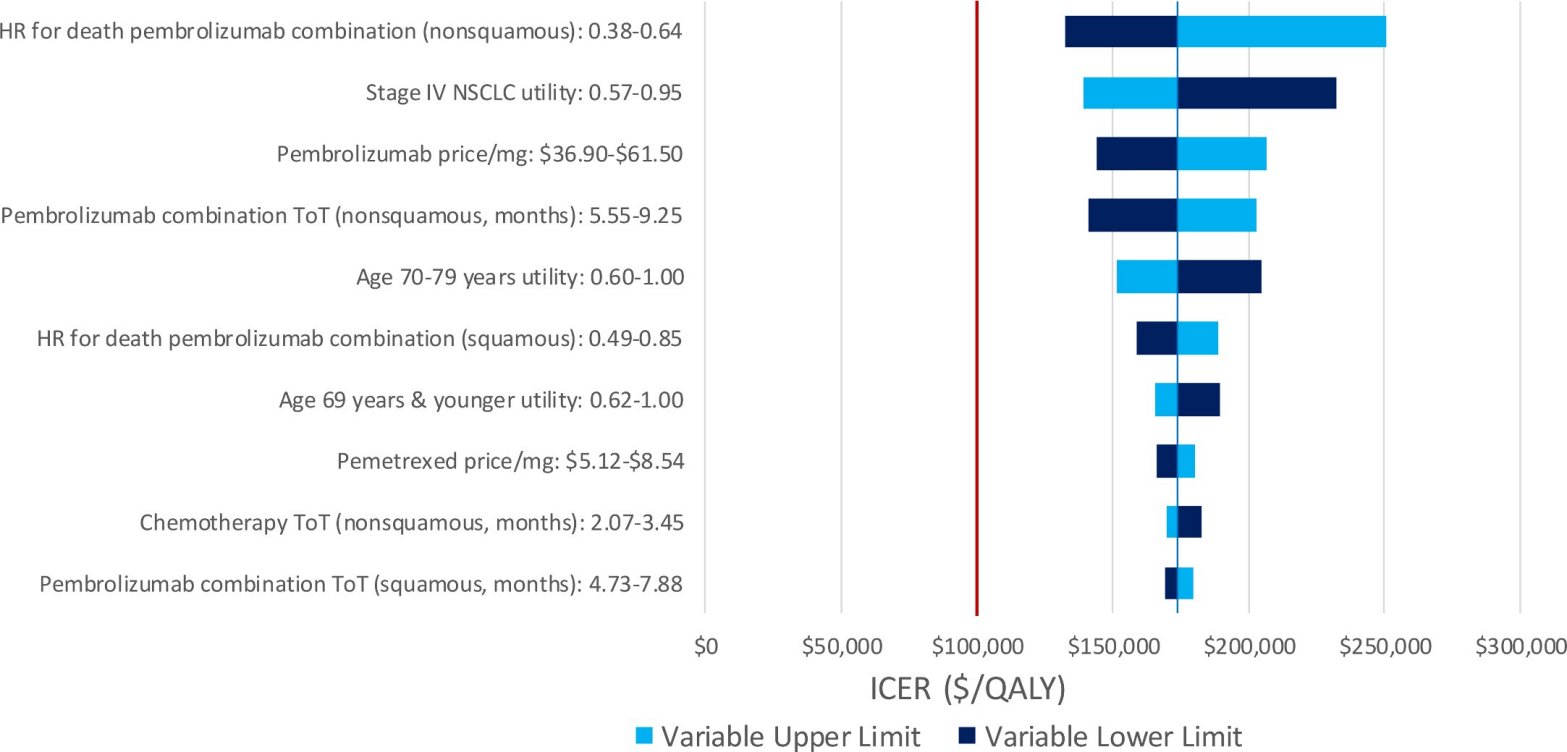
Deterministic sensitivity analysis for the pembrolizumab combination therapy model—pembrolizumab combination versus chemotherapy. HR, hazard ratio; NSCLC, non-small cell lung cancer; ToT, time on treatment; OS, overall survival.

**Fig 3:**
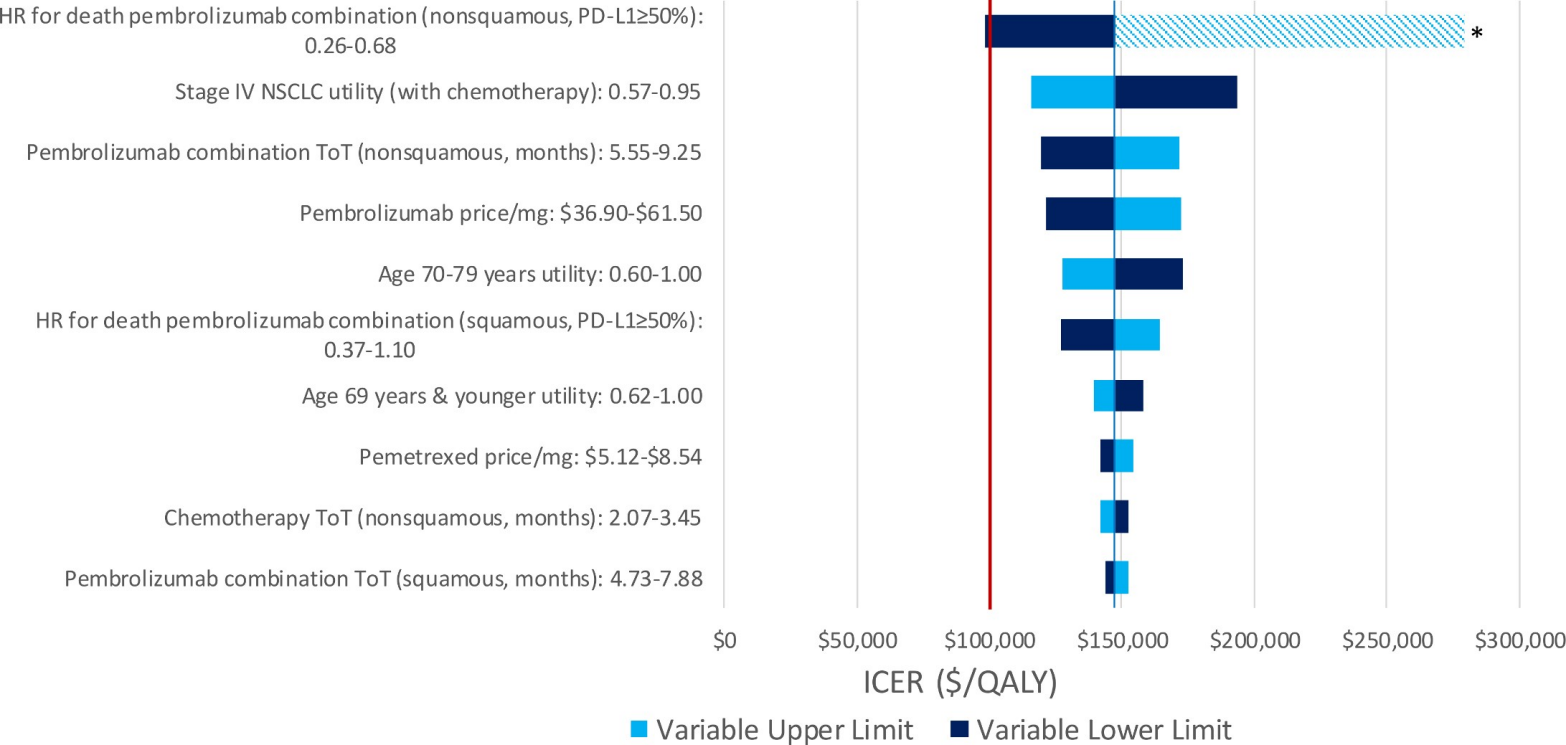
Deterministic sensitivity analysis for the PD-L1-high model—pembrolizumab combination versus chemotherapy. HR, hazard ratio; NSCLC, non-small cell lung cancer; ToT, time on treatment; OS, overall survival * Pembrolizumab combination therapy did not weakly dominate pembrolizumab monotherapy, therefore, the efficient frontier changed order and pembrolizumab combination no longer had an ICER compared to chemotherapy.

Pembrolizumab combination therapy was not cost-effective in any of the tested ranges in the one-way deterministic sensitivity analyses for the pembrolizumab combination therapy model. For the PD-L1-high model, pembrolizumab combination therapy was cost-effective at the lower limit of the hazard ratio for death for patients on pembrolizumab combination with nonsquamous histology and PD-L1 expression of at least 50% but was not cost-effective in any other scenarios. Pembrolizumab monotherapy was weakly dominated in most cases, with exceptions for the upper limit of the hazard ratio for death for patients on pembrolizumab combination with nonsquamous histology and PD-L1 expression of at least 50% (pembrolizumab monotherapy ICER of $177,869/QALY versus chemotherapy) and the lower limit of the hazard ratio for death for patients on pembrolizumab monotherapy (pembrolizumab monotherapy ICER of $122,474/QALY versus chemotherapy). Results for the 10 inputs with greatest magnitude of effect are shown in Fig 2 for the pembrolizumab combination therapy model and Fig 3 for the PD-L1-high model.

## Discussion

To estimate the cost-effectiveness of two prominent pembrolizumab treatment regimens for stage IV NSCLC patients with varying burden and types of comorbidities, we developed microsimulation models that compared treatment strategies using pembrolizumab combination therapy, pembrolizumab monotherapy, or chemotherapy alone. Even when evaluated in a patient population with Charlson score equal to 0, the ICER for pembrolizumab combination therapy was well above the WTP threshold of $100,000/QALY in both models. Cost-effectiveness worsened slightly (ICERs in both models increased by approximately 1%) when these treatments were evaluated in Charlson 1 patients and even more so (ICERs in both models increased by approximately 5%) when evaluated in Charlson 2+ patients. Patient populations with congestive heart failure or cerebrovascular disease had ICERs greater than those for patients with a Charlson score of 2+. In the PD-L1-high model, the pembrolizumab monotherapy arm was weakly dominated by the pembrolizumab combination therapy arm, even with the improved quality of life from avoiding chemotherapy in the first line of treatment.

Despite significantly improving survival for stage IV NSCLC patients compared to chemotherapy alone (hazard ratio = 0.49 for pembrolizumab combination therapy in nonsquamous patients; hazard ratio = 0.64 for pembrolizumab combination therapy in squamous patients; hazard ratio = 0.63 for pembrolizumab monotherapy) [3–5], pembrolizumab treatment regimens were not cost-effective in any of the patient populations evaluated. In the current literature and based on results from RCTs, ICERs for both pembrolizumab regimens have been estimated to be around $100,000/QALY [10–12], One of the drivers that led to these treatment strategies not being cost-effective in our analysis was the shorter OS of patients in a real-world setting captured by SEER-Medicare data, compared to the academic clinical trial setting in which the RCTs were conducted. Median OS in the stage IV NSCLC population with Charlson score equal to 0 receiving chemotherapy were 7.5 months for nonsquamous patients and 7.1 months for squamous patients, as determined by our analysis of SEER-Medicare survival data. In the RCTs for pembrolizumab combination therapy, median OS was 11.3 months for both nonsquamous and squamous patients on chemotherapy [4, 5]. which indicates the potential for healthy volunteer effect. In the RCT for pembrolizumab monotherapy, median OS of patients on chemotherapy even reached 14.2 months [3]. With shorter underlying survival times for patients in the real-world setting, even the improvements in survival rate shown in the RCTs did not lead to high enough incremental survival benefit to offset the cost of these therapies. In patient populations with significant comorbidities, survival times decreased further, while costs of treatment decreased more modestly, leading to the higher ICERs presented in our results.

Given that only a small percentage of patients in the U.S. are treated in academic centers [16], our cost-effectiveness results point to the importance of reasonable drug pricing. In the case of Charlson 0 patients with no PD-L1 expression cutoff, pembrolizumab combination therapy would require a pembrolizumab price reduction of 60% to become cost-effective. With the ICERs for these treatment regimens increasing as comorbidity level increases, even greater price reductions would be necessary to attain cost-effectiveness in comorbid populations. Our results suggest that the effectiveness of pembrolizumab in the general population of stage IV NSCLC patients, and especially so in the population with significant comorbidities, does not justify its current listed price, as it is based on clinical results in an unrepresentative sample of healthier patients.

By modeling these pembrolizumab-based treatment regimens in a real-world setting represented by the SEER-Medicare population, we can see that the current literature on pembrolizumab does not provide a realistic assessment of its cost-effectiveness. In order to get a more accurate evaluation of the true effectiveness of pembrolizumab in the general stage IV NSCLC patient population, more data need to be published on the survival of patients in realistic clinical settings, not just academic clinical trials. Once these data are available, a justifiable price point can be more robustly determined.

### Limitations

We made several assumptions in the development of our models. First, our analysis uses the hazard ratios for death from RCTs to estimate the death rates for potential patients receiving pembrolizumab treatments [3, 4]. Since patients with significant comorbidities have largely been excluded from RCTs for these treatments, the survival of these patients is unknown. Thus, we assumed that patients with comorbidities gain a similar relative survival benefit over chemotherapy as do patients in RCTs. Second, model utilities were sourced from the literature and represent the general experience of patients with advanced stage NSCLC. Third, SEER-Medicare data is available up to 2015 and, therefore, does not allow us to evaluate these indications using data that reflect the adoption of second-line immunotherapy in 2015. Utilizing updated SEER-Medicare data in this way is the focus of future analyses. Finally, the RCT for pembrolizumab combination therapy experienced crossover from the chemotherapy group to the pembrolizumab group of 41.3% and the RCT for pembrolizumab monotherapy experienced crossover of 43.7%, potentially confounding the hazard ratios for death between pembrolizumab and chemotherapy [4]. Adjusting for crossover would likely increase the effectiveness of the pembrolizumab arm (lower hazard ratio for death), as was seen in the RCT for pembrolizumab monotherapy (0.63 unadjusted hazard ratio for death versus 0.49 hazard ratio for death after crossover adjustment) [3]. However, the effect of a lower hazard ratio for death was studied in the deterministic sensitivity analysis (Figs 2 and 3).

## Conclusions

For patients with varying burden and types of comorbidities, first-line stage IV NSCLC treatment with pembrolizumab does not represent a cost-effective strategy compared to standard-of-care chemotherapy. The quality-adjusted survival benefit that can be gained through pembrolizumab treatment does not justify current pricing and, as the prevalence of immunotherapy treatment grows, more attention should be focused on determining reasonable price targets that may make these treatments more affordable. Additionally, resources should be focused on collecting data for and performing clinical trials with more representative NSCLC patient populations in order to understand the true survival benefit of immunotherapies, which would allow for a more accurate estimate of their cost-effectiveness.

## Supporting information

S1 File Supporting information tables. (DOCX)

S2 File Data. (XLSX)
