# Supplementary material for: Cost-effectiveness of pembrolizumab for advanced non-small cell lung cancer patients with varying comorbidity burden
Source: PLoS One. 2020 Jan 29;15(1):e0228288. doi: 10.1371/journal.pone.0228288 (PMC6988966; doi:10.1371/journal.pone.0228288)
Supplement: S1 File — (DOCX) [file pone.0228288.s001.docx]

**Supporting Information**

**Contents:**

S1 Table A. Treatment Parameters

S2 Table B. SEER-Medicare Primary Data Analysis Summary

S3 Table C. Cost and Utility Parameters

References

**Table A. Treatment Parameters**

| **Variable** | **Input Value** | **Source** |
| --- | --- | --- |
| **Dose Sizes** | | |
| Pembrolizumab monotherapy | 200 mg fixed | Reck, et al., 2016 [1] |
| Pembrolizumab + Carboplatin + Pemetrexed | pembrolizumab 200 mg fixed;  carboplatin, AUC 6.0 mg/ml/min; pemetrexed, 500 mg/m^2^ | Gandhi, et al., 2018 [2] |
| Pembrolizumab + Carboplatin + Paclitaxel | pembrolizumab 200 mg fixed;  carboplatin, AUC 6.0 mg/ml/min; paclitaxel, 200 mg/m^2^ | Paz-Ares, et al., 2018 [3] |
| Pembrolizumab + Carboplatin + Nab-Paclitaxel | pembrolizumab 200 mg fixed;  carboplatin, AUC 6.0 mg/ml/min;  nab-paclitaxel, 100 mg/m^2^ | Paz-Ares, et al., 2018 [3] |
| Carboplatin + Pemetrexed | carboplatin, AUC 6.0 mg/ml/min;  pemetrexed, 500 mg/m^2^ | Gandhi, et al., 2018 [2] |
| Carboplatin + Paclitaxel | carboplatin, AUC 6.0 mg/ml/min;  paclitaxel, 200 mg/m^2^ | Paz-Ares, et al., 2018 [3] |
| Docetaxel | 75 mg/m^2^ | Herbst, et al., 2016 [4] |
| **Infusion Timing** | | |
| Pembrolizumab monotherapy | 3 weeks up to 35 cycles | Reck, et al., 2016 [1] |
| Pembrolizumab + Carboplatin + Pemetrexed | 3 weeks up to 4 cycles;  pembrolizumab up to 35 cycles; pemetrexed only thereafter | Gandhi, et al., 2018 [2] |
| Pembrolizumab + Carboplatin + Paclitaxel | 3 weeks up to 4 cycles;  pembrolizumab up to 35 cycles | Paz-Ares, et al., 2018 [3] |
| Pembrolizumab + Carboplatin + Nab-Paclitaxel | 3 weeks up to 4 cycles  (nab-paclitaxel weekly);  pembrolizumab up to 35 cycles | Paz-Ares, et al., 2018 [3] |
| Carboplatin + Pemetrexed | 3 weeks up to 4 cycles;  pemetrexed only thereafter | Gandhi, et al., 2018 [2] |
| Carboplatin + Paclitaxel | 3 weeks up to 4 cycles | Paz-Ares, et al., 2018 [3] |
| Docetaxel | 3 weeks | Herbst, et al., 2016 [4] |
| **Patient Stratification** | | |
| Squamous histology | 25% (Avg. Males 30%, Females 20%) | Meza, et al., 2015 [5] |
| Nonsquamous histology | 75% | Meza, et al., 2015 [5] |
| **Subsequent Therapy Assumptions** | | |
| Pembrolizumab monotherapy | 60% of patients receive second-line therapy | Lazzari, et al., 2017 [6] |
| Pembrolizumab combination | 30.5% of patients receive second-line therapy | Gandhi, et al., 2018 [2] |
| Chemotherapy | 46.6% of patients receive second-line therapy | Gandhi, et al., 2018 [2] |

**Table B. SEER-Medicare Primary Data Analysis for Stage IV NSCLC**

| **Patient Summary Table** | |
| --- | --- |
| Total patients in analysis (number) | 16,492 |
| Median Age (years) | 72 |
| Males (number / %) | 9,556 / 57.9% |
| **Histology** | |
| Nonsquamous (number / %) | 12,452 / 75.5% |
| Squamous (number / %) | 4,040 / 24.5% |
| **Comorbidity Level** | |
| Charlson score = 0 (number / %) | 7,446 / 45.1% |
| Charlson score = 1 (number / %) | 4,512 / 27.4% |
| Charlson score = 2+ (number / %) | 3,163 / 19.2% |
| Charlson score not identifiable (number / %) | 1,371 / 8.3% |
| **Comorbidity Type** | |
| Chronic obstructive pulmonary disease (number / %) | 4,306 / 26.1% |
| Diabetes (number / %) | 2,751 / 16.7% |
| Peripheral vascular disease (number / %) | 1,343 / 8.1% |
| Congestive heart failure (number / %) | 1,036 / 6.3% |
| Cerebrovascular disease (number / %) | 844 / 5.1% |
| Comorbidity type not identifiable (number / %) | 1,371 / 8.3% |

**Table C. Cost and Utility Parameters**

| **Variable** | **Input Value** | **Source** |
| --- | --- | --- |
| **Costs (2019 USD)** | | |
| Pembrolizumab price per mg | $49.20 | CMS 2019 ASP Drug Pricing Files |
| Pemetrexed price per mg | $6.83 | CMS 2019 ASP Drug Pricing Files |
| Carboplatin price per mg | $0.06 | CMS 2019 ASP Drug Pricing Files |
| Docetaxel price per mg | $1.16 | CMS 2019 ASP Drug Pricing Files |
| Paclitaxel price per mg | $0.15 | CMS 2019 ASP Drug Pricing Files |
| Nab-paclitaxel (protein bound) per mg | $11.85 | CMS 2019 ASP Drug Pricing Files |
| Imaging/Surveillance | $1,408.77 | CMS.gov [7], CPT 78816 |
| Drug administration per hour | $143.08 | CMS.gov [7], CPT 96413 |
| Immunohistochemical test | $108.48 | CMS.gov [7], CPT 88342 |
| Monthly best supportive care (regression estimate for 70 year old) | $637 | SEER-Medicare regression analysis |
| Death costs  (regression estimate for 70 year old) | $9,433 | SEER-Medicare regression analysis |
| Adverse events (pembrolizumab monotherapy) | $886 | hcupnet.ahrq.gov; Reck, et al., 2016 [1] |
| Adverse events (pembrolizumab + carboplatin + pemetrexed) | $4,779 | hcupnet.ahrq.gov; Gandhi, et al., 2018 [2] |
| Adverse events (pembrolizumab + carboplatin + paclitaxel) | $6,795 | hcupnet.ahrq.gov; Paz-Ares, et al., 2018 [3] |
| Adverse events (carboplatin + pemetrexed) | $3,911 | hcupnet.ahrq.gov; Gandhi, et al., 2018 [2] |
| Adverse events (carboplatin + paclitaxel) | $7,634 | hcupnet.ahrq.gov; Paz-Ares, et al., 2018 [3] |
| Adverse events (docetaxel) | $2,439 | hcupnet.ahrq.gov; Herbst, et al., 2016 [4] |
| **Utilities** | | |
| Stage IV NSCLC  (with chemotherapy) | 0.76 | Tramontano, et al., 2015 [8] |
| Stage IV NSCLC  (without chemotherapy) | 0.91 | Assumption based on clinical input (20% improvement over utility with chemotherapy) |
| Age Utility (69 years & younger) | 0.83 | Hanmer, et al., 2006 [9] (National HRQoL Tables – 2003; average of males & females) |
| Age Utility (70-79 years) | 0.80 | Hanmer, et al., 2006 [9] (National HRQoL Tables – 2003; average of males & females) |
| Age Utility (80 years & older) | 0.75 | Hanmer, et al., 2006 [9] (National HRQoL Tables – 2003; average of males & females) |

**References**

1. Reck M, Rodriguez-Abreu D, Robinson AG, Hui R, Csoszi T, Fulop A, et al. Pembrolizumab versus Chemotherapy for PD-L1-Positive Non-Small-Cell Lung Cancer. N Engl J Med. 2016;375(19):1823-33. doi: 10.1056/NEJMoa1606774. PubMed PMID: 27718847.

2. Gandhi L, Rodriguez-Abreu D, Gadgeel S, Esteban E, Felip E, De Angelis F, et al. Pembrolizumab plus Chemotherapy in Metastatic Non-Small-Cell Lung Cancer. N Engl J Med. 2018. doi: 10.1056/NEJMoa1801005. PubMed PMID: 29658856.

3. Paz-Ares L, Luft A, Vicente D, Tafreshi A, Gumus M, Mazieres J, et al. Pembrolizumab plus Chemotherapy for Squamous Non-Small-Cell Lung Cancer. N Engl J Med. 2018;379(21):2040-51. doi: 10.1056/NEJMoa1810865. PubMed PMID: 30280635.

4. Herbst RS, Baas P, Kim DW, Felip E, Perez-Gracia JL, Han JY, et al. Pembrolizumab versus docetaxel for previously treated, PD-L1-positive, advanced non-small-cell lung cancer (KEYNOTE-010): a randomised controlled trial. Lancet. 2016;387(10027):1540-50. doi: 10.1016/S0140-6736(15)01281-7. PubMed PMID: 26712084.

5. Meza R, Meernik C, Jeon J, Cote ML. Lung cancer incidence trends by gender, race and histology in the United States, 1973-2010. PLoS One. 2015;10(3):e0121323. doi: 10.1371/journal.pone.0121323. PubMed PMID: 25822850; PubMed Central PMCID: PMCPMC4379166.

6. Lazzari C, Bulotta A, Ducceschi M, Vigano MG, Brioschi E, Corti F, et al. Historical Evolution of Second-Line Therapy in Non-Small Cell Lung Cancer. Front Med (Lausanne). 2017;4:4. doi: 10.3389/fmed.2017.00004. PubMed PMID: 28168189; PubMed Central PMCID: PMCPMC5253463.

7. Physician Fee Schedule Search [Internet]. Centers for Medicare & Medicaid Services. 2018. Available from: <https://www.cms.gov/apps/physician-fee-schedule/search/search-criteria.aspx>.

8. Tramontano AC, Schrag DL, Malin JK, Miller MC, Weeks JC, Swan JS, et al. Catalog and comparison of societal preferences (utilities) for lung cancer health states: results from the Cancer Care Outcomes Research and Surveillance (CanCORS) study. Med Decis Making. 2015;35(3):371-87. doi: 10.1177/0272989X15570364. PubMed PMID: 25670839.

9. Hanmer J, Lawrence WF, Anderson JP, Kaplan RM, Fryback DG. Report of nationally representative values for the noninstitutionalized US adult population for 7 health-related quality-of-life scores. Med Decis Making. 2006;26(4):391-400. doi: 10.1177/0272989X06290497. PubMed PMID: 16855127.
